# Targeted literature review of current treatments and unmet need in moderate rheumatoid arthritis in the United Kingdom

**DOI:** 10.1093/rheumatology/keab464

**Published:** 2021-06-03

**Authors:** Peter C Taylor, Matthew Woods, Catherine Rycroft, Priya Patel, Sophee Blanthorn-Hazell, Toby Kent, Marwan Bukhari

**Affiliations:** 1 Nuffield Department of Orthopaedics, Rheumatology and Musculoskeletal Sciences, University of Oxford, Oxford; 2 BresMed Health Solutions Ltd, Sheffield; 3 AbbVie Ltd, Maidenhead; 4 Department of Rheumatology, Royal Lancaster Infirmary, Lancaster, UK

**Keywords:** rheumatoid arthritis, moderate, unmet need, clinical burden, economic burden, treatment

## Abstract

**Objectives:**

The burden and treatment landscape of RA is poorly understood. This research aimed to identify evidence on quality of life, caregiver burden, economic burden, treatment patterns and clinical outcomes for patients with moderate RA in the United Kingdom.

**Methods:**

A systematic literature review was performed across multiple databases and screened against pre-defined inclusion criteria.

**Results:**

A total of 2610 records were screened; seven studies presenting evidence for moderate RA were included. These patients were found to incur substantial burden, with moderate to severe levels of disability. Compared with patients in remission, moderate RA patients reported higher levels of disability and decreased EQ-5D utility scores. The majority of patients did not feel that their current therapy adequately controlled their disease or provided sufficient symptom relief. In the United Kingdom, the National Institute for Health and Care Excellence (NICE) have not approved advanced therapies (such as biological disease-modifying anti-rheumatic drugs) for patients with moderate disease, which restricts access for these patients.

**Conclusion:**

The evidence available on the burden of moderate RA is limited. Despite current treatments, moderate RA still has a substantial negative impact, given that a DAS28 disease activity score defined as being in the moderate range does not qualify them for access to advanced therapies in the United Kingdom. For these patients, there is a particular need for further studies that investigate their burden and the impact of treating them earlier. Such information would help guide future treatment decisions and ensure the most effective use of resources to gain the best outcomes for patients with moderate RA.

**Rheumatology key messages**

- The impact of persistent moderately active rheumatoid arthritis is poorly understood, with limited evidence available.
- Available evidence indicates that moderately active rheumatoid arthritis incurs a substantial disease burden.
- Access to advanced therapies in the United Kingdom is restricted for moderate rheumatoid arthritis patients.

## Introduction

RA, an autoimmune disease and the most common form of inflammatory polyarthritis [1, 2], affects ∼400 000 people in the United Kingdom. Symptoms include fatigue, depression and swollen, stiff and painful joints. Left untreated or undermanaged, RA is characterized by chronic pain, disability, and a 32% excess risk of mortality compared with people of the same age who do not have RA [3]. RA causes joint damage in 80–85% of patients, with particularly rapid damage occurring during the first 2 years of the disease [4], highlighting a need for early treatment.

Although RA currently has no cure, the treatment landscape includes disease-modifying anti-rheumatic drugs (DMARDs) to reduce systemic and local inflammation. With reduced inflammation, symptoms improve, structural damage to joints is delayed, and function is preserved. Other treatments include plain analgesics and non-steroidal anti-inflammatory drugs, which target pain but do not prevent the patient’s joints from being eroded by RA.

In the United Kingdom, patients are eligible for different treatments based on their disease activity, defined by the 28-joint disease activity score (DAS28). Patients with moderate RA receive conventional synthetic DMARDs (csDMARDs). Those with severe RA, who have poor response to a combination of csDMARDs, are eligible for advanced therapies [5]; biological DMARDs (bDMARDs), such as anti-TNF monoclonal antibodies, and small-molecule targeted synthetic DMARDs (tsDMARDs), such as Janus kinase (JAK) inhibitors.

The burden and treatment landscape of RA in the United Kingdom is poorly understood, particularly for patients with moderate RA whose therapeutic options are limited. This literature review identifies evidence for the management of moderate and severe RA in the United Kingdom, the impact on healthcare resource use and costs, symptoms, patient and caregiver quality of life (QoL), disease progression and treatment outcomes.

## Methods

Comprehensive, targeted literature searches were performed on 17 October 2018 across multiple literature databases using a combination of MeSH^®^ and free-text terms for RA, disease severity, treatment patterns, QoL, caregiver burden, economic burden and clinical outcomes (Supplementary Tables S1–S3, available at *Rheumatology* online). Websites were searched for conference proceedings and clinical guidelines. Manual bibliography searches of included studies and review of sources identified by the authors and clinical experts were also performed. To identify the latest relevant evidence for the United Kingdom, database searches were restricted to English-language publications, published within the previous 5 years (from August 2013), conducted in humans; conference searches were limited to abstracts published from 2016.

Title and abstracts and full text screening were performed by one researcher, as per the predefined inclusion criteria (Table 1). Any uncertainty was judged by a second researcher. Relevant data were extracted, stratified by moderate, moderate to severe, and severe disease (see the Supplementary Material, available at *Rheumatology* online, for additional detail).

**Table 1:** Eligibility criteria for inclusion in the targeted literature review

Criteria	Description
Population	Patients with moderate or severe RA
Topics of interest	Treatment guidelines Costs and healthcare resource use Humanistic burden (patient-reported outcomes and quality of life) Caregiver burden Treatment patterns Real-world clinical outcomes^a^ (including evidence within real-world clinical practice from observational studies, retrospective studies, database studies, registries and clinical audits)
Interventions	Searches for real-world clinical data were restricted to the following interventions^b^: Conventional synthetic DMARDs (gold injections, hydroxychloroquine, leflunomide, methotrexate, sulfasalazine, azathioprine) Biological DMARDs (adalimumab, certolizumab pegol, etanercept, infliximab, abatacept, rituximab, tocilizumab, golimumab) JAK inhibitors (upadacitinib, tofacitinib, baricitinib, filgotinib)
Country	Restricted to UK studies^c^

DMARDs: disease-modifying anti-rheumatic drugs; JAK: Janus kinase.

a The searches for real-world evidence studies were not restricted by outcomes, and all relevant studies were included.

b Interventions included evidence for biosimilars, where available.

c The database searches were not restricted by country of interest to avoid missing any potentially relevant studies due to issues with referencing in the databases. Instead, the restriction to focus the review on UK studies was applied during screening.

## Results

### Overview of findings

**Fig. 1:**
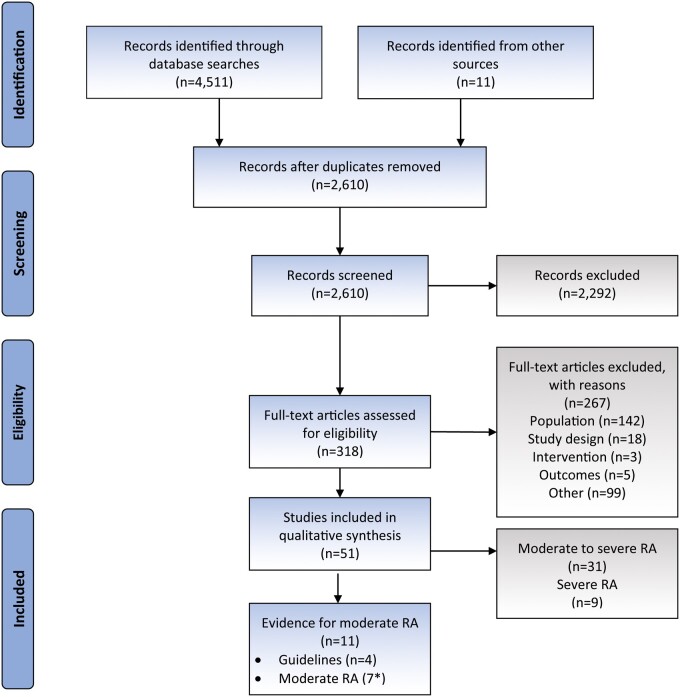
PRISMA flow diagram *includes three studies that presented evidence separately for moderate RA patients within a study of moderate to severe RA. Exclusion reason of ‘Population’ includes studies in patients other than those with RA; exclusion reason ‘Study design’ includes studies that did not report information for the topics of interest based on their design (e.g. *in vitro* studies or commentaries); exclusion reason ‘Intervention’ includes studies investigating non-pharmaceutical interventions; exclusion reason ‘Outcomes’ includes studies that did not present evidence for any of the topics of interest; exclusion reason ‘Other’ includes studies with no UK data or abstracts published before 2016. Adapted from Moher *et al.*, 2009 [6]. PRISMA, Preferred Reporting Items for Systemic Reviews and Meta-Analyses.

Fig. 1 presents the Preferred Reporting Items for Systematic Reviews and Meta-Analyses flowchart [6]. A total of 2610 records were retrieved from the literature databases and screened; 51 met the predefined inclusion criteria. Of these 51 references, four were included from the manual review. Table 2 presents a breakdown of the included topic areas alongside the number of identified references for moderate, severe, and a mixed population of moderate and severe RA. Several publications provided evidence for more than one topic of interest. Most studies (*n* = 38) focussed on patients with moderate or severe disease, with data not split by disease severity. Nine articles focussed on severe RA and four on moderate RA. In addition, of the articles that reported a mixed population, three reported results separately by disease severity.

**Table 2:** Summary of papers identified by topic and disease severity

Topic area	Total	Moderate RA	Severe RA	Mixed moderate/severe RA
Treatment guidelines	4	0	0	4
Cost and healthcare resource use	6	0	2	4^a^
Humanistic burden	24	4	2	18^b^
Caregiver burden	1	1	0	0
Treatment patterns	29	2	8	19
Real-world clinical outcomes	28	3	8	17
Total^c^	51	4	9	38^d^

a Including one where data were analysed by severity. ^b^Including two where data were analysed by severity. ^c^Some articles provided evidence for more than one topic area. ^d^Including three where data were analysed by severity.

### Summary of identified literature

A key finding of the review was that evidence regarding the unmet need for patients with moderate RA is limited while that for moderate to severe and severe RA is better understood. Therefore, this article focusses on the evidence identified for moderate RA; Table 3 presents the included moderate RA studies, along with the topics reported within each. The findings for moderate to severe and severe RA are summarized in the Supplementary Material, available at *Rheumatology* online.

**Table 3:** Studies presenting evidence for moderate RA

Reference	Treatment guidelines	Cost and healthcare resource use	Humanistic burden	Caregiver burden	Treatment patterns	Real-world clinical outcomes
NICE, 2018 [5]	✓					
SIGN, 2011 [7]	✓					
BSR/BHPR; Ledingham *et al.* 2017 [8]	✓					
EULAR; Smolen *et al.* 2019 [9]	✓					
Bergstra *et al.* 2018^a^ [11]		✓				
Kotak *et al.* 2015 [12]			✓		✓	✓
Prothero *et al.* 2016 [13]			✓	✓		
Scott *et al.* 2018 [14]			✓			✓
Gullick *et al.* 2016 [15]			✓		✓	✓
Mian *et al.* 2016^a^ [16]			✓			
Nikiphorou *et al.* 2016^a^ [17]			✓			
	Section 4.2.1	Section 4.2.2	Section 4.2.3	Section 4.2.4	Section 4.2.5	Section 4.2.6

a Study presented evidence for moderate RA patients within a study of moderate to severe RA.

### Summary of treatment guidelines

Key treatment guidelines commonly used by UK clinicians are provided by the National Institute for Health and Care Excellence (NICE) [5], the Scottish Intercollegiate Guidelines Network (SIGN) [7], the British Society for Rheumatology/British Health Professionals in Rheumatology (BSR/BHPR) [8] and the EULAR [9]. The guidelines from NICE and SIGN are generally consistent, both providing the following key recommendations [5, 7]:

- csDMARDs as first-line therapy (i.e. methotrexate and sulfasalazine; NICE also recommends another option, leflunomide); and
- bDMARDs (adalimumab, etanercept, infliximab, certolizumab pegol, golimumab, abatacept and tocilizumab) when disease activity score DAS28 is >5.1 (i.e. severe disease) and patients display a lack of response to intensive csDMARDs.

The SIGN guidelines differ from NICE recommendations by suggesting a combination DMARD strategy, rather than sequential monotherapy, in patients with an inadequate response to initial DMARD therapy [7]. The BSR/BHPR guidelines have not been updated since 2010 [8].

The EULAR guidelines were last updated in December 2019 [9]. This update specifies methotrexate as first-line therapy, or leflunomide or sulfasalazine where methotrexate is contraindicated. Like SIGN, EULAR recommends combination therapy with csDMARDs and advanced therapies to achieve treatment targets [9]. If the target is not achieved, other csDMARDs should be used, or advanced therapies added if poor prognostic factors are present. The two key changes to the recommendations since 2016 are [10]:

- the preference of bDMARDs over tsDMARDs, including JAK inhibitors, was revised, placing tsDMARDs alongside bDMARDs in the treatment pathway; and
- for patients not fully responsive to a csDMARD and with poor prognostic factors, the previous recommendation—to ‘consider adding’ a bDMARD or tsDMARD—is replaced by the stronger recommendation ‘to add’ a bDMARD or tsDMARD (current practice would be to start with a bDMARD).

### Cost and healthcare resource use

Six studies were identified reporting evidence for costs and healthcare resource use: one considered a mixed RA population and presented data separately for patients with moderate RA; three considered use in patients with moderate to severe RA; two considered use in patients with severe RA. The data for patients with moderate RA are presented here; moderate to severe and severe data are summarized in the Supplementary Material, available at *Rheumatology* online.

Bergstra *et al.* (2018) [11] reported the relative lack of access to advanced therapies in the moderate RA population in the United Kingdom, which achieved the lowest clinical criteria score for access to bDMARDs (a composite score taking into account prescription and reimbursement rules) across the 12 included comparator countries [the United States (state of Massachusetts), Mexico, South Africa, Japan, Brazil, the United Kingdom, Spain, Ireland, Portugal, France, India (state of Maharashtra) and the Netherlands]. The United Kingdom was also reported to only have 14.7% of patients using bDMARDs (across the overall RA population); the lowest across all included countries (Spain 16.3%, the Netherlands 28.2%, Portugal 44.5%, the United States 48.6%, Japan 50.5%, France 60.2% and Ireland 75.0%). Their analysis suggested that this lower UK bDMARD usage rate may be due to relatively strict prescription and reimbursement rules in the United Kingdom.

The study also reported a correlation between Gross Domestic Product (GDP) per capita and DAS28 remission. However, while the United Kingdom has a relatively high GDP per capita, it had one of the lowest percentages of patients in DAS28 remission (26.0%), most likely related to the low use of bDMARDs. Indeed, a statistically significant relationship between bDMARD use and patients in DAS28 remission was identified.

### Humanistic burden of disease

Twenty-four studies were identified presenting evidence on the humanistic burden of RA: four moderate, 18 moderate to severe and two severe RA. Two of the mixed population studies presented data separately by disease severity; the evidence for patients with moderate RA is presented here; the moderate to severe and severe evidence is summarized in the Supplementary Material, available at *Rheumatology* online.

In a retrospective study using the BSR Biologics Register for RA (BSRBR-RA), comparing patients with moderate RA treated either with etanercept or csDMARDs, those treated with etanercept had significantly higher disease activity at the time treatment was initiated [12]. At baseline, the patients treated with etanercept were significantly more likely to be unemployed because of disability (33% *vs* 16%), had a longer disease duration (14.1 *vs* 10.2 years), numerically higher DAS28 score (4.6 *vs* 4.4), a higher level of disability [HAQ Disability Index (HAQ-DI) 1.9 *vs* 1.5], and a poorer health-related QoL (HRQL), as measured by the 36-item Short Form Health Survey physical component score (27.3 *vs* 29.8), than patients treated with csDMARDs (*P* <0.001 for all). However, these patients had significantly reduced disease activity and better HRQL after 6, 12 and 24 months of treatment compared with patients receiving csDMARD therapy.

One study used a series of patient interviews to investigate the importance of self-care in patients with moderate RA [13], reporting that patients experienced frustration and even depression as a result of their condition. The majority of patients (six out of nine) did not feel their treatment regimen adequately controlled their disease or provided sufficient symptom relief. However, as a result, they were more open to trying intensive management (by addition of advanced therapies to concomitant csDMARDs). Patients hoped that intensive management would improve their physical symptoms through reduced pain, improved mobility and increased independence.

The other studies in patients with moderate RA were broadly consistent, with HAQ-DI scores ranging from 1.0 to 1.4, indicating moderate to severe levels of disability [11, 14–17]. One study reported that compared with patients in remission, HAQ-DI scores in patients with moderate RA were increased by 1.06, and EQ-5D scores were reduced by 0.27 [15]. HAQ-DI scores were found to progress at a significantly faster rate in all DAS28 categories (including for patients with moderate RA) compared with patients in remission [17]; as DAS28 scores increased (even within disease severity categories), HAQ-DI scores also increased, as did the rate of HAQ-DI progression [17].

### Caregiver burden of disease

Limited evidence was found reporting the burden for caregivers of patients with RA: one study in moderate RA and no studies in the moderate to severe or severe populations. Caregivers were concerned by the lack of continuity of care for patients, and their main hope was that access to intensive management strategies would increase independence and improve mobility [13]. However, caregivers’ views were affected by how stable patients were with their current treatment. Basically, caregivers felt stability would be lost when treatments changed.

### Treatment patterns

A total of 29 studies presented evidence on treatment patterns: two for moderate, 19 for moderate to severe, and eight for severe RA. The evidence on treatment patterns presented within the moderate studies was extremely limited; therefore, the evidence for the overall RA population is summarized here, focussing on patients with moderate to severe RA.

The studies show that the use of csDMARDs has increased dramatically over time. Researchers report up to 87% of patients have received csDMARDs [16] (70–87%) [16, 18, 19], with methotrexate the most commonly used treatment (60–88%) [18–20]. The use of combination csDMARDs has also increased, although the wide range of reported results across the included studies makes it difficult to identify the leading treatment (24–76.3%) [16, 19, 21].

The use of bDMARDs has also increased, although not to the same extent as csDMARDs. In a study of patients who had been initiated on advanced therapies between 2009 and 2010, 23% of patients received only monotherapy with no concomitant csDMARD [20]. A UK-wide audit of patients with RA between May 2012 and December 2015 showed only 4% received advanced therapies, most commonly etanercept [22]. However, only 36% of patents eligible for advanced therapies actually received them [22].

Steroid use was found to vary widely across the United Kingdom (Table 3 for moderate RA; Supplementary Material, available at *Rheumatology* online, for moderate to severe and severe RA). A single study was identified in patients with moderate RA, reporting that 23% of patients received steroids [15]. In patients with moderate to severe RA, steroid use was largely unchanged between 1996 and 2014, staying at ∼12% despite increased use of csDMARDs [16]. Another study of csDMARDs in patients with moderate to severe RA, undertaken in a single centre, found an extremely high number of patients receiving steroids: 51–65% at baseline rising to 67–78% by the end of 12 months of treatment [21].

### Real-world clinical outcomes

A total of 28 publications were identified that presented evidence on real-world clinical outcomes: three for moderate, 17 for moderate to severe, and eight for severe RA. A summary of key outcomes for the identified studies is presented in Table 4 for moderate RA and in Supplementary Table S4, available at *Rheumatology* online, for moderate to severe and severe RA.

**Table 4:** Summary of real-world clinical outcomes in moderate RA

Study	Study type [*n*]	Treatment	Remission	Response	Steroid use	DAS28	HAQ-DI	EQ-5D
Moderate RA								
Kotak, 2015 [12]	Retrospective cohort study [*n* = 1754]	bDMARDS (including etanercept, infliximab, and adalimumab) (*n* = 211)	12 months: 26.5% 24 months: 27.5%	24 months, EULAR: Good: 41.7% Moderate: 22.3% None: 36.0%	—	Mean (s.d.): change at 24 months: −1.1 (0.14)	—	—
		csDMARDs (*n* = 1543)	12 months: 16.9% 24 months: 20.3%	24 months, EULAR: Good: 29.9% Moderate: 20.5% None: 49.6%	—	Mean (s.d.): change at 24 months: −0.59 (0.04)	—	—
Gullick, 2016 [15]	10-year prospective observational study [*n* = 1693]	‘Intensive treatment’ with csDMARDs, often in combination, and a range of bDMARDs (as per standard practice)	2005: 18% 2015: 27%	No response (high disease activity): 2005: 25% 2015: 16%	23%	Mean: 2005: 4.1 2015: 3.7	Mean: 2005: 1.26 2015: 1.15	Mean: 2005: 0.47 2015: 0.56
Scott, 2018 [14]	Retrospective database study [*n* = 208]	Treat to target; treatments not specified (bDMARD naïve at baseline)	—	—	—	—	Mean: Baseline: 1.21 Change <0.22 units at 12 months: 43%	—

bDMARD: biologic DMARD; cs DMARD: conventional synthetic DMARD; DAS28: 28-joint disease activity score; DMARD: disease-modifying anti-rheumatic drug; EQ-5D: EuroQol 5-dimensions; HAQ-DI: HAQ Disability Index.

A significantly greater percentage of patients receiving etanercept (bDMARD) compared with those receiving csDMARDs achieved remission by 6 months (22.4% *vs* 13.4%, respectively; *P* < 0.05), 12 months (26.5% *vs* 16.9%, respectively; *P* < 0.05), and 24 months (27.5% *vs* 20.3%, respectively; *P* < 0.05), despite a significantly higher disease activity at the time of etanercept initiation [12]. A significantly greater percentage of patients receiving etanercept also achieved a low disease activity status (DAS28 2.6–3.2) compared with patients receiving csDMARDs at 6 months (15.2% *vs* 12.6%, respectively; *P* < 0.05) and 24 months (19.4% *vs* 15.2%, respectively; *P* < 0.05). A significantly smaller proportion of patients had no change in disease activity, no EULAR response, or experienced disease progression when treated with etanercept compared with csDMARDs (all *P* < 0.05).

A prospective cohort study evaluated disease activity and outcomes at a single centre aiming to treat to a target DAS28 < 2.6(16), reporting that remission increased from 18% to 27% after a 10-year follow-up. Mean DAS28 and HAQ-DI scores and the proportion of patients with high disease scores (i.e. severe disease) decreased. However, 22% of patients had persistently high disease activity despite treatment with csDMARDs, often in combination with each other, and a range of advanced therapies. Only 9% of patients with persistent high disease activity were receiving advanced therapies, compared with 18–20% of other groups (*P* = 0.034). Patients with RA, persistent moderate disease activity and a moderate disability also failed to reach the treat-to-target goal of remission/low disease activity [14]. These patients continued to have moderate disease and persistent moderate disability even after 12 months of treatment.

## Discussion

Current treatment guidelines are generally aligned, at least when it comes to the use of csDMARDs. According to NICE, csDMARDs (methotrexate, sulfasalazine or leflunomide hydroxychloroquine) are recommended for patients with newly diagnosed RA (as first-line therapy). As RA progresses, treatment options are generally dependent on treatment response and disease severity. Initially, increased csDMARD therapy is recommended, but bDMARDs (typically an anti-TNF initially) may be used where strict criteria are met, including severe disease activity (DAS28 > 5.1) and lack of response to csDMARD combinations. The 2019 update to the EULAR guidelines has also now moved tsDMARDs, which include JAK inhibitors, alongside bDMARDs in the treatment pathway. Here, the notable difference from NICE is that the recommended threshold of disease activity to access advanced therapies is set at DAS28 > 3.2. These strict criteria recommended by NICE may explain why the use of advanced therapies in patients with moderate RA is so low in the United Kingdom, despite the continued and substantial burden of disease these patients experience and that their disease may not be properly controlled using conventional therapy. The evidence indicates a lack of consistency in treatment for patients in the United Kingdom and a significant proportion of patients not receiving appropriate treatment. Widening access to advanced therapies, including JAK inhibitors and biologic therapies, for patients with moderate disease in the United Kingdom would meet a key unmet need in RA. This assumption is supported by a recent UK study [23] that concluded that patients with moderate RA may benefit from more aggressive, advanced therapy. However, the costs of bDMARDs would need to be reduced by nearly 50% to be considered a cost-effective use of National Health Service (NHS) resources, according to a recent NICE multiple technology appraisal assessing the cost-effectiveness of bDMARDs in patients with moderate RA [24]. The introduction of cheaper biosimilar products, which are anticipated to be cost-effective in moderate RA, and therefore more likely to be available on the NHS for these patients, may lead to a change in the UK reimbursement landscape for moderate RA.

For patients with moderate disease, RA has been shown to incur a substantial burden in terms of healthcare resource use and costs, increased absenteeism and presenteeism, as well as the humanistic burden for both patients and their caregivers, although evidence on caregiver burden is extremely limited. Even with existing treatment options, a substantial proportion of patients remain outside of disease remission. Although use of advanced therapies does improve outcomes, there is still a large unmet need for patients with moderate disease, who have limited access to these therapies. Increasing access to these more effective therapies and encouraging access earlier in the disease course may help to reduce the burden and improve outcomes for patients with moderate RA.

Despite having what are considered more acceptable DAS28 disease scores, patients with moderate disease still face substantial burdens, with patients stating a desire for reduced pain, better mobility and increased independence. These patients were shown to have significant levels of disability and poor HRQL, a significant level of unemployment due to disability (16–33%), and a prolonged period of time with disease symptoms (10.2–14.1 years). After 6, 12 and 24 months of treatment with advanced therapies, however, these patients had significantly reduced disease activity and better HRQL compared with patients receiving csDMARD therapy, which highlights the substantial humanistic burden for patients with moderate RA and the importance of advanced therapies in helping to reduce this burden. The evidence identified also highlights the importance of providing detailed information on medications to both patients and their caregivers to put their minds at ease. However, there is a clear paucity of evidence available specific to the moderate RA population, particularly for fatigue, pain and limitations of activities, which are shown to be key drivers of the patient burden for the more widely reported ‘moderate to severe’ population. Thus, there is a need for more evidence to better understand the substantial burden in the moderate RA population and help drive treatment decisions in the future.

The use of steroids remains controversial due to the well-documented risks of treatment, including cardiovascular morbidity, infection and osteoporosis. The evidence on steroid use for the overall RA patient population (only one study reported data for moderate patients) varies between studies, between treatment centres and across regions, which makes it difficult to understand the full extent of the associated burden in UK clinical practice. Additionally, this variability is also likely due to the difficulties in accurately capturing steroid use for these patients. However, from the available evidence, there is still a substantial proportion of patients with RA receiving steroid treatment alongside their csDMARD therapy and, despite advances in available and effective targeted therapies, there is the potential that this burden is still extremely high. It is probable that the high disease activity threshold set by NICE for access to targeted therapies is the driver for the ongoing use of steroids in an effort to better control symptoms despite the associated longer-term risks. These points highlight the pressing need for the use of safe and effective treatments to reduce steroid use in these patients.

As with all literature reviews, both the current review and the identified data have certain limitations. First, the database searches were limited to evidence published within the past 5 years. While this ensured that only the most recent—and therefore most relevant—data were found, it does leave the potential for omitting older evidence that may still have provided useful information. Second, given the nature of the studies themselves, the literature identified in this type of review is often heterogeneous, making comparisons between the studies or synthesis of the data challenging. Finally, in areas where evidence is limited, such as in this review and particularly for patients with moderate RA, the evidence base is often restricted to small numbers of studies that report different outcomes or present evidence in different ways, which makes it difficult to check for consistency across results and ensure that an accurate view of the evidence base is being presented.

Although the evidence available on the burden of RA is limited, particularly for moderate disease, there appears to be a significant impact on patients, even with advances in treatment options. This impact is most apparent in patients with RA whose DAS28 disease activity does not qualify them for access to advanced therapies but who still face a substantial disease burden. For the relatively poorly understood moderate RA population in particular, who are not currently eligible for advanced therapies in the United Kingdom, there is a need for further studies that investigate the burden for these patients and the impact of treating them earlier. Such information would help guide treatment decisions in the future and ensure the most effective use of resources to gain the best outcomes for patients.

## Supplementary Material

keab464_Supplementary_Data
