# Supplementary material for: Targeted literature review of current treatments and unmet need in moderate rheumatoid arthritis in the United Kingdom
Source: Rheumatology (Oxford). 2021 Jun 3;60(11):4972–81. doi: 10.1093/rheumatology/keab464 (PMC8566217; doi:10.1093/rheumatology/keab464)
Supplement: keab464_Supplementary_Data [file keab464_supplementary_data.zip › keab464-suppl_data/rhe-20-2821-File003.docx]

# Supplementary Material

## 1.1 Targeted literature review methods

### 1.1.1 Literature search

Comprehensive, targeted literature searches were performed on 17 October 2018 within MEDLINE^®^ (via Embase.com), EMBASE^®^ (via Embase.com), MEDLINE In-Process (via PubMed), EconLit^®^ (via EBSCO), and The Cochrane Library (Cochrane Database of Systematic Reviews; Cochrane Central Register of Controlled Trials; Cochrane Clinical Answers) using a combination of MeSH^®^ and free-text terms for rheumatoid arthritis (RA) coupled with terms for moderate or severe disease and for treatment patterns, quality of life (QoL), caregiver burden, economic burden, clinical outcomes, and adherence.

To identify the most recent and relevant evidence, searches were restricted to English-language publications, articles published within the previous 5 years (from August 2013), and studies conducted in humans. Comments, letters, editorials, and case reports were excluded. The full search strategies and results from each of the databases are presented below.

Table S1. Search strategy for Embase via Embase.com (search date: 17 October 2018)

|  | # | Search terms | Hits |
| --- | --- | --- | --- |
| Population | #1 | ‘arthritis, rheumatoid’/mj OR (‘arthritis’:ti AND ‘rheumatoid’:ti) OR ‘rheumatoid arthritis’:ti | 120,321 |
|  | #2 | 'moderate':ab,ti OR 'severe':ab,ti OR ('das28':ab,ti AND '5.1':ab,ti) | 1,587,769 |
|  | #3 | #1 AND #2 | 11,429 |
| Treatment patterns | #4 | (‘Clinical practice’/mj OR ‘treatment pattern*’:ab,ti OR ‘prescribing pattern*’:ab,ti OR ((‘Retrospective Studies’/mj OR ‘Cohort Studies’/mj OR ‘Observational Study’:it OR ‘Observational Study’/mj OR observational*:ab,ti OR ‘cohort stud*’:ab,ti OR retrospective:ab,ti OR registry:ab,ti OR database:ab,ti OR audit:ab,ti) AND (‘Therapeutics’/mj OR ‘therapy’:lnk OR treatment*:ti OR treat:ti OR treated:ti OR treating:ti OR therap*:ti))) NOT (‘clinical trial’:it OR ‘clinical trials’/mj OR ‘clinical trial’:ti OR ‘clinical trial, phase ii’:it OR ‘clinical trials, phase ii’/mj OR ‘clinical trial, phase iii’:it OR ‘phase 2 clinical trial’:ti OR ‘clinical trials, phase iii’/mj OR ‘phase 3 clinical trial’:ti OR ‘Review’:it OR ‘Review Literature’/mj OR ‘literature review':ti OR ‘Meta-Analysis’:it OR ‘Meta-Analysis’/mj OR ‘systematic review’:ti OR ‘systematic literature review’:ti OR ‘meta-analysis’:ab,ti OR ‘meta analysis’:ab,ti) | 363,543 |
| Adherence | #5 | (‘Treatment Adherence and Compliance’/mj OR ‘Medication Adherence’/mj OR ‘Patient Compliance’/mj OR complian*:ab,ti OR adheren*:ab,ti OR persisten*:ab,ti) NOT (‘Meta-Analysis’:it OR ‘Meta-Analysis’/mj OR ‘systematic review’:ti OR ‘systematic literature review’:ti OR ‘meta-analysis’:ab,ti OR ‘meta analysis’:ab,ti) | 733,424 |
| Quality of life and patient activities (including caregiver burden) | #6 | (‘Quality of Life’/mj OR ‘quality of life’:ti OR ‘quality-of-life’:ti OR QoL:ti OR hrqol:ti OR hqol:ab,ti OR ‘patient report*’:ab,ti OR ‘patient-report*’:ab,ti OR ‘Questionnaires’/mj OR ‘Self Report’/mj OR questionnaire*:ab,ti OR ‘Health Status Indicators’/mj OR ‘Activities of Daily Living’/mj OR ‘Personal Autonomy’/mj OR ‘Self Care’/mj OR ‘self care’:ab,ti OR ‘self-care’:ab,ti OR ‘limitation of activit*’:ab,ti OR autonomy:ab,ti OR ‘activities of daily living’:ab,ti OR ‘functional status’:ab,ti OR ‘physical function*’:ab,ti OR ‘Caregivers’/mj OR carer*:ab,ti OR caregiver*:ab,ti OR ‘care giver*’:ab,ti OR ‘care-giver*’:ab,ti OR societal:ab,ti OR ‘Patient Preference’/mj OR ‘Patient Satisfaction’/mj OR satisfaction:ab,ti OR satisfied:ab,ti OR preference*:ab,ti OR prefer*:ab,ti OR ‘Focus Groups’/mj OR ‘Interview’:it OR ‘Interviews’/mj OR ‘Absenteeism’/mj OR ‘Sick Leave’/mj OR productivity:ab,ti OR ‘absenteeism’:ab,ti OR ‘presenteeism’:ab,ti OR ‘sick leave’:ab,ti OR ‘sick day*’:ab,ti OR ‘disability leave’:ab,ti OR ‘work loss’:ab,ti OR ‘signs and symptoms’/mj OR ‘symptom assessment’/mj) NOT ((‘Review’:it OR ‘Review Literature’/exp OR ‘literature review’:ti) NOT (‘Meta-Analysis’:it OR ‘Meta-Analysis’/mj OR ‘systematic review’:ti OR ‘systematic literature review’:ti OR ‘meta-analysis’:ab,ti OR ‘meta analysis’:ab,ti)) | 1,533,617 |
| Economic burden | #7 | (‘Cost of Illness’/mj OR ‘Health Expenditures’/mj OR ‘Economics, hospital’/mj OR ‘Economics, medical’/mj OR ‘Economics, nursing’/mj OR ‘Economics, pharmaceutical’/mj OR ‘Health Resources/utilization’/mj OR ‘Fees and Charges’/mj OR ‘Health Care Costs’/mj OR cost:ti OR costs:ti OR economic*:ti OR pharmacoeconomic*:ti OR resource:ti OR resources:ti) NOT (‘Cost-Benefit Analysis’/mj OR ‘Models, Economic’/mj OR model*:ti OR ‘cost-effective*’:ti OR ‘cost effective*’:ti OR ‘cost-utility’:ti OR ‘cost utility’:ti OR ‘Review’:it OR ‘Review Literature’/mj OR ‘literature review’:ti) NOT (‘Meta-Analysis’:it OR ‘Meta-Analysis’/mj OR ‘systematic review’:ti OR ‘systematic literature review’:ti OR ‘meta-analysis’:ab,ti OR ‘meta analysis’:ab,ti) | 200,670 |
| Clinical outcomes – observational and real-world studies | #8 | (‘Retrospective Studies’/mj OR ‘Cohort Studies’/mj OR ‘Observational Study’:it OR ‘Observational Study’/mj OR observational*:ab,ti OR ‘cohort stud*’:ab,ti OR retrospective:ab,ti OR ‘registries’/mj OR ‘registry’:ab,ti OR ‘database’:ab,ti OR ‘audit’:ab,ti) OR (‘real world’:ab,ti OR ‘real-world’:ab,ti) OR (‘SDAI’:ab,ti OR ‘simple disease activity index’:ab,ti OR ‘CDAI’:ab,ti OR ‘clinical disease activity index’:ab,ti) NOT (‘clinical trial’:it OR ‘clinical trials’/mj OR ‘clinical trial’:ti OR ‘clinical trial, phase ii’:it OR ‘clinical trials, phase ii’/mj OR ‘clinical trial, phase iii’:it OR ‘phase 2 clinical trial’:ti OR ‘clinical trials, phase iii’/mj OR ‘phase 3 clinical trial’:ti OR ‘Review’:it OR ‘Review Literature’/mj OR ‘literature review’:ti OR ‘Meta-Analysis’:it OR ‘Meta-Analysis’/mj OR ‘systematic review’:ti OR ‘systematic literature review’:ti OR ‘meta-analysis’:ab,ti OR ‘meta analysis’:ab,ti) | 1,408,972 |
|  | #9 | #3 AND (#4 OR #5 OR #6 OR #7 OR #8) | 4,171 |
| Exclusion terms – exclude animals | #10 | ‘Animals’/exp NOT ‘Humans’/exp | 5,121,527 |
| Exclusion terms – study type | #11 | ‘Comment’:it OR ‘Letter’:it OR ‘Editorial’:it OR ‘Case Reports’:it OR ‘case study’:ab,ti OR ‘case studies’:ab,ti OR ‘case report’:ab,ti OR ‘case reports’:ab,ti | 2,139,396 |
|  | #12 | #9 NOT (#10 OR #11) | 4,120 |
| Limit to English language | #13 | #12 AND [english]/lim | 3,939 |
| Limit to past 5 years | #14 | #13 AND [1-8-2013]/sd NOT [1-1-2019]/sd | 2,497 |

Table S2. Search strategy for Cochrane via The Cochrane library (search date: 17 October 2018)

|  | # | Search terms | Hits |
| --- | --- | --- | --- |
| Population | #1 | [mh "arthritis, rheumatoid" [mj]] OR ("arthritis":ti AND "rheumatoid":ti) OR "rheumatoid arthritis":ti | 8,392 |
|  | #2 | “moderate”:ti,ab OR “severe”:ti,ab OR ("DAS28":ti,ab AND ("5.1":ti,ab OR ("5.1":ti,ab AND “3.2”:ti,ab) OR (3.2 near/2 5.1):ti,ab OR (“<=5.1”:ti,ab AND “>3.2”:ti,ab) OR (“≤5.1”:ti,ab AND “>3.2”:ti,ab))) | 125,361 |
|  | #3 | #1 AND #2 | 1,312 |
| Treatment patterns | #4 | ([mh "Physician's Practice Patterns" [mj]] OR treatment pattern*:ti,ab OR prescribing pattern*:ti,ab OR (([mh "Retrospective Studies" [mj]] OR [mh “Cohort Studies" [mj]] OR "Observational Study":PT OR [mh "Observational Studies as Topic" [mj]] OR observational*:ti,ab OR cohort stud*:ti,ab OR retrospective:ti,ab OR "registry":ti,ab OR “database”:ti,ab OR “audit”:ti,ab) AND ([mh "Therapeutics" [mj]] OR [mh /TH] OR treatment*:ti OR treat:ti OR treated:ti OR treating:ti OR therap*:ti))) NOT ("clinical trial":PT OR [mh "clinical trials as topic" [mj]] OR "clinical trial":ti OR "clinical trial, phase ii":PT OR [mh "clinical trials, phase ii as topic" [mj]] OR "clinical trial, phase iii":PT OR "phase 2 clinical trial":ti OR [mh "clinical trials, phase iii as topic" [mj]] OR "phase 3 clinical trial":ti OR "Review":PT OR [mh "Review Literature as Topic" [mj]] OR “literature review”:ti OR "Meta-Analysis":PT OR [mh "Meta-Analysis as Topic" [mj]] OR "systematic review”:ti OR “systematic literature review”:ti OR “meta-analysis”:ti,ab OR “meta analysis”:ti,ab) | 24,970 |
| Adherence | #5 | ([mh "Treatment Adherence and Compliance" [mj]] OR [mh "Medication Adherence" [mj]] OR [mh "Patient Compliance" [mj]] OR complian*:ti,ab OR adheren*:ti,ab OR persisten*:ti,ab) NOT ("Meta-Analysis":PT OR [mh "Meta-Analysis as Topic" [mj]] OR “systematic review”:ti OR “systematic literature review”:ti OR “metaanalysis”:ti,ab OR “meta analysis”:ti,ab) | 58,144 |
| Quality of life and patient activities (including caregiver burden) | #6 | ([mh “Quality of Life” [mj]] OR “quality of life”:ti OR QoL:ti OR hrqol:ti OR hqol:ti,ab OR “patient report*”:ti,ab OR (patient NEAR/2 report*):ti,ab OR [mh “Questionnaires” [mj]] OR [mh “Self Report” [mj]] OR questionnaire*:ti,ab OR [mh “Health Status Indicators”[mj]] OR [mh “Activities of Daily Living” [mj]] OR [mh “Personal Autonomy” [mj]] OR [mh “Self Care” [mj]] OR “self care”:ti,ab OR (self NEAR/2 care):ti,ab OR limitation of activit*:ti,ab OR autonomy:ti,ab OR “activities of daily living”:ti,ab OR “functional status”:ti,ab OR physical function*:ti,ab OR [mh “Caregivers” [mj]] OR carer*:ti,ab OR caregiver*:ti,ab OR care giver*:ti,ab OR (care NEAR/2 giver*):ti,ab OR societal:ti,ab OR [mh “Patient Preference” [mj]] OR [mh “Patient Satisfaction” [mj]] OR satisfaction:ti,ab OR satisfied:ti,ab OR preference*:ti,ab OR prefer*:ti,ab OR [mh “Focus Groups” [mj]] OR “Interview”:PT OR [mh “Interviews as Topic” [mj]] OR [mh “Absenteeism” [mj]] OR [mh “Sick Leave” [mj]] OR productivity:ti,ab OR “absenteeism”:ti,ab OR “presenteeism”:ti,ab OR “sick leave”:ti,ab OR sick day*:ti,ab OR “disability leave”:ti,ab OR “work loss”:ti,ab OR [mh “signs and symptoms” [mj]] OR [mh “symptom assessment” [mj]]) NOT (“Review”:PT OR [mh “Review Literature as Topic”] OR “literature review”:ti) NOT (“Meta Analysis”:PT OR [mh “Meta Analysis as Topic” [mj]] OR “systematic review”:ti OR “systematic literature review”:ti OR “meta analysis”:ti,ab) | 85,691 |
| Economic burden | #7 | ([mh "Cost of Illness" [mj]] OR [mh "Health Expenditures" [mj]] OR [mh “Economics, hospital” [mj]] OR [mh "Economics, medical" [mj]] OR [mh "Economics, nursing" [mj]] OR [mh “Economics, pharmaceutical” [mj]] OR [mh "Health Resources" [mj]] OR [mh "Fees and Charges" [mj]] OR [mh "Health Care Costs" [mj]] OR cost:ti OR costs:ti OR economic*:ti OR pharmacoeconomic*:ti OR resource:ti OR resources:ti) NOT ([mh "Cost Benefit Analysis" [mj]] OR [mh "Models, Economic" [mj]] OR model*:ti OR (cost near/2 effective*):ti OR cost effective*:ti OR (cost NEAR/2 utility):ti OR “cost utility”:ti OR "Review":PT OR [mh "Review Literature as Topic"] OR “literature review”:ti) NOT ("Meta Analysis":PT OR [mh "Meta Analysis as Topic" [mj]] OR “systematic review”:ti OR “systematic literature review”:ti OR “meta analysis”:ti,ab) | 7,514 |
| Clinical outcomes – observational and real-world studies | #8 | ([mh “Retrospective Studies” [mj]] OR [mh “Cohort Studies” [mj]] OR “Observational Study”:PT OR [mh “Observational Study as Topic" [mj]] OR observational*:ti,ab OR cohort stud*:ti,ab OR retrospective:ti,ab OR [mh "registries" [mj]] OR “registry”:ti,ab OR “database”:ti,ab OR “audit”:ti,ab) OR (“real world”:ti,ab OR “real-world”:ti,ab) OR (“SDAI”:ti,ab OR “simple disease activity index”:ti,ab OR “CDAI”:ti,ab OR “clinical disease activity index”:ti,ab) NOT (“clinical trial”:PT OR [mh “clinical trials as topic” [mj]] OR “clinical trial”:ti OR “clinical trial, phase ii”:PT OR [mh “clinical trials, phase ii as topic” [mj]] OR “clinical trial, phase iii”:PT OR “phase 2 clinical trial”:ti OR [mh “clinical trials, phase iii as topic” [mj]] OR “phase 3 clinical trial”:ti OR “Review”:PT OR [mh “Review Literature as Topic" [mj]] OR “literature review”:ti OR “Meta Analysis”:PT OR [mh “Meta Analysis as Topic” [mj]] OR “systematic review”:ti OR “systematic literature review”:ti OR “meta analysis”:ti,ab) | 80,800 |
|  | #9 | #3 AND (#4 OR #5 OR #6 OR #7 OR #8) | 385 |
| Exclusion terms – exclude animals | #10 | [mh Animals] NOT [mh Humans] | 0 |
| Exclusion terms – study type | #11 | “Comment”:PT OR “Letter”:PT OR “Editorial”:PT OR “Case Reports”:PT OR “case study”:ti,ab OR “case studies”:ti,ab OR “case report”:ti,ab OR “case reports”:ti,ab | 14,040 |
|  | #12 | #9 NOT (#10 OR #11) | 384 |
| Limit to English language | #13 | #12 with Cochrane Library publication date from Aug 2013 to Dec 2018 | 301 |
| Limit to past 5 years | #14 | #13 with Cochrane Library publication date from Aug 2013 to Dec 2018, in Cochrane Reviews, Cochrane Protocols, Trials and Clinical Answers | 301 |

Table S3. Search strategy for Medline-in-Process via PubMed (search date: 17 October 2018)

|  | # | Search terms | Hits |
| --- | --- | --- | --- |
| Population | #1 | "arthritis, rheumatoid"[Majr] OR ("arthritis"[Title] AND "rheumatoid"[Title]) OR "rheumatoid arthritis"[Title] | 94,864 |
|  | #2 | “moderate”[Title/Abstract] OR “severe”[Title/Abstract] OR ("DAS28"[Title/Abstract] AND ("5.1"[Title/Abstract] OR ("5.1"[Title/Abstract] AND “3.2”[Title/Abstract]) OR “3.2-5.1”[Title/Abstract] OR “3.2 - 5.1”[Title/Abstract] OR (“<=5.1”[Title/Abstract] AND “>3.2”[Title/Abstract]) OR (“≤5.1”[Title/Abstract] AND “>3.2”[Title/Abstract]))) | 1,130,844 |
|  | #3 | #1 AND #2 | 7,089 |
| Treatment patterns | #4 | ("Physician's Practice Patterns"[Majr] OR treatment pattern*[Title/Abstract] OR prescribing pattern*[Title/Abstract] OR (("Retrospective Studies"[Majr] OR "Cohort Studies"[Majr] OR "Observational Study"[Publication Type] OR "Observational Study as Topic"[Majr] OR observational*[Title/Abstract] OR cohort stud*[Title/Abstract] OR retrospective[Title/Abstract] OR "registry"[Title/Abstract] OR “database”[Title/Abstract] OR “audit”[Title/Abstract]) AND ("Therapeutics"[Majr] OR "therapy"[Subheading] OR treatment*[Title] OR treat[Title] OR treated[Title] OR treating[Title] OR therap*[Title]))) NOT ("clinical trial"[Publication Type] OR "clinical trials as topic"[Majr Terms] OR "clinical trial"[Title] OR "clinical trial, phase ii"[Publication Type] OR "clinical trials, phase ii as topic"[Majr Terms] OR "clinical trial, phase iii"[Publication Type] OR "phase 2 clinical trial"[Title] OR "clinical trials, phase iii as topic"[Majr Terms] OR "phase 3 clinical trial"[Title] OR "Review"[Publication Type] OR "Review Literature as Topic"[Majr] OR “literature review”[Title] OR "Meta-Analysis"[Publication Type] OR "Meta-Analysis as Topic"[Majr] OR "systematic review”[Title] OR “systematic literature review”[Title] OR “meta-analysis”[Title/Abstract] OR “meta analysis”[Title/Abstract]) | 394,167 |
| Adherence | #5 | ("Treatment Adherence and Compliance"[Majr] OR "Medication Adherence"[Majr] OR "Patient Compliance"[Majr] OR complian*[Title/Abstract] OR adheren*[Title/Abstract] OR persisten*[Title/Abstract]) NOT ("Meta-Analysis"[Publication Type] OR "Meta-Analysis as Topic"[Majr] OR "systematic review”[Title] OR “systematic literature review”[Title] OR “meta-analysis”[Title/Abstract] OR “meta analysis”[Title/Abstract) | 597,575 |
| Quality of life and patient activities (including caregiver burden) | #6 | ("Quality of Life"[Majr] OR "quality of life"[Title] OR "quality-of-life"[Title] OR QoL[Title] OR hrqol[Title] OR hqol[Title/Abstract] OR patient report*[Title/Abstract] OR patient-report*[Title/Abstract] OR "Questionnaires"[Majr] OR "Self Report"[Majr] OR questionnaire *[Title/Abstract] OR "Health Status Indicators"[Majr] OR "Activities of Daily Living"[Majr] OR "Personal Autonomy"[Majr] OR "Self Care"[Majr] OR “self care ”[Title/Abstract] OR “self-care”[Title/Abstract] OR limitation of activit*[Title/Abstract] OR autonomy[Title/Abstract] OR “activities of daily living”[Title/Abstract] OR "functional status"[Title/Abstract] OR physical function*[Title/Abstract] OR "Caregivers"[Majr] OR carer*[Title/Abstract] OR caregiver*[Title/Abstract] OR care giver*[Title/Abstract] OR care-giver*[Title/Abstract] OR societal[Title/Abstract] OR "Patient Preference"[Majr] OR "Patient Satisfaction"[Majr] OR satisfaction[Title/Abstract] OR satisfied[Title/Abstract] OR preference*[Title/Abstract] OR prefer*[Title/Abstract] OR "Focus Groups"[Majr] OR "Interview"[Publication Type] OR "Interviews as Topic"[Majr] OR "Absenteeism"[Majr] OR "Sick Leave"[Majr] OR productivity[Title/Abstract] OR “absenteeism”[Title/Abstract] OR "presenteeism"[Title/Abstract] OR “sick leave”[Title/Abstract] OR sick day*[Title/Abstract] OR “disability leave”[Title/Abstract] OR “work loss”[Title/Abstract] OR “signs and symptoms”[Majr] OR “symptom assessment”[Majr]) NOT (("Review"[Publication Type] OR "Review Literature as Topic"[Mesh] OR “literature review”[Title]) NOT ("Meta-Analysis"[Publication Type] OR "Meta-Analysis as Topic"[Majr] OR "systematic review”[Title] OR “systematic literature review”[Title] OR “meta-analysis”[Title/Abstract] OR “meta analysis”[Title/Abstract])) | 1,983,697 |
| Economic burden | #7 | ("Cost of Illness"[Majr] OR "Health Expenditures"[Majr] OR “Economics, hospital”[Majr] OR "Economics, medical"[Majr] OR "Economics, nursing"[Majr] OR “Economics, pharmaceutical”[Majr] OR "Health Resources/utilization"[Majr] OR "Fees and Charges"[Majr] OR "Health Care Costs"[Majr] OR cost[Title] OR costs[Title] OR economic*[Title] OR pharmacoeconomic*[Title] OR resource[Title] OR resources[Title]) NOT ("Cost-Benefit Analysis"[Majr] OR "Models, Economic"[Majr] OR model*[Title] OR cost-effective*[Title] OR cost effective*[Title] OR “cost-utility”[Title] OR “cost utility”[Title] OR "Review"[Publication Type] OR "Review Literature as Topic"[Mesh] OR “literature review”[Title]) NOT ("Meta-Analysis"[Publication Type] OR "Meta-Analysis as Topic"[Majr] OR "systematic review”[Title] OR “systematic literature review”[Title] OR “meta-analysis”[Title/Abstract] OR “meta analysis”[Title/Abstract])) | 173,199 |
| Clinical outcomes – observational and real-world studies | #8 | ("Retrospective Studies"[Majr] OR "Cohort Studies"[Majr] OR "Observational Study"[Publication Type] OR "Observational Study as Topic"[Majr] OR observational*[Title/Abstract] OR cohort stud*[Title/Abstract] OR retrospective[Title/Abstract] OR "registries"[Majr] OR "registry"[Title/Abstract] OR “database”[Title/Abstract] OR “audit”[Title/Abstract]) OR ("real world"[Title/Abstract] OR "real-world"[Title/Abstract]) OR (“SDAI”[Title/Abstract] OR “simple disease activity index”[Title/Abstract] OR “CDAI”[Title/Abstract] OR “clinical disease activity index”[Title/Abstract]) NOT ("clinical trial"[Publication Type] OR "clinical trials as topic"[Majr] OR "clinical trial"[Title] OR "clinical trial, phase ii"[Publication Type] OR "clinical trials, phase ii as topic"[Majr] OR "clinical trial, phase iii"[Publication Type] OR "phase 2 clinical trial"[Title] OR "clinical trials, phase iii as topic"[Majr] OR "phase 3 clinical trial"[Title] OR "Review"[Publication Type] OR "Review Literature as Topic"[Majr] OR “literature review”[Title] OR "Meta-Analysis"[Publication Type] OR "Meta-Analysis as Topic"[Majr] OR "systematic review”[Title] OR “systematic literature review”[Title] OR “meta-analysis”[Title/Abstract] OR “meta analysis”[Title/Abstract]) | 885,130 |
|  | #9 | #3 AND (#4 OR #5 OR #6 OR #7 OR #8) | 1,869 |
| Exclusion terms – exclude animals | #10 | "Animals"[MeSH] NOT "Humans"[MeSH] | 4,505,605 |
| Exclusion terms – study type | #11 | "Comment"[Publication Type] OR "Letter"[Publication Type] OR "Editorial"[Publication Type] OR "Case Reports"[Publication Type] OR "case study"[Title/Abstract] OR "case studies"[Title/Abstract] OR “case report”[Title/Abstract] OR “case reports”[Title/Abstract] | 3,523,887 |
|  | #12 | #9 NOT (#10 OR #11) | 1,713 |
| Limit to publication status ahead of print | #13 | (#12 AND (pubstatusaheadofprint OR inprocess[sb])) | 86 |
|  | #14 | (((publisher[sb] NOT pubstatusnihms NOT pubstatuspmcsd NOT pmcbook) OR (pubstatusaheadofprint))) | 446,867 |
|  | #15 | (#12 AND #14) | 37 |
|  | #16 | (#13 OR #15) | 89 |

### 1.1.2 Conference searches, additional website searches, and manual searches

Additional websites were searched to obtain recent information that may not yet have been published in full-text journal articles. Conference searches were limited to abstracts published from 2016 onwards. These searches included the British Society of Rheumatology (BSR) Conference, the Annual European Congress of Rheumatology and the American College of Rheumatology Conference. In addition, any relevant conference abstracts from 2016 onwards referenced in Embase were included as part of the database search.

Additional website searches included the Turning Research into Practice database for clinical guidelines. Manual searches of bibliographies of included studies, as well as review of potential sources of interest identified by the authors and clinical experts, were also performed.

### 1.1.3 Study selection and data extraction

Identified titles and abstracts of full-text articles, abstracts from conference proceedings, and clinical and treatment guidelines were screened by one researcher in the initial review for relevance to the topic, as per the predefined inclusion/exclusion criteria. For the secondary review, the shortlisted full-text articles were retrieved and screened against the same inclusion/exclusion criteria by one researcher. Any uncertainty about the inclusion and extraction of studies was checked and judged by a second senior researcher. Reasons for study exclusions were recorded.

Relevant data were extracted from each identified publication that met the predefined inclusion/exclusion criteria and tabulated in summary tables. Extracted data included citation, study objective, patient numbers, current treatment patterns for patients with moderate and severe RA in the United Kingdom and how these patterns affect disease progression, treatment outcomes, patient symptoms, patient and caregiver QoL, and healthcare resource use and costs. Evidence was stratified by moderate, moderate to severe, and severe disease, where possible.

## 1.2 Evidence for moderate to severe and severe RA

### 1.2.1 Cost and healthcare resource use

#### Moderate to severe and severe RA

The studies identified in this review indicated that RA is associated with substantial healthcare resource use and costs. A retrospective medical chart review(19) reported that these costs included hospitalizations (6.8% of patients) and physician visits (mean of 4.5 visits/year), physiotherapy (44.7% of patients), occupational therapy (30.1% of patients), assistive devices (10.7% of patients), procedures (43.7% of patients), and ongoing monitoring, including liver function tests (mean of 6.5 visits/year) and C-reactive protein tests (mean of 5.2 tests/year). The level of intervention and support required highlights the lack of effective treatment and the associated unmet need for patients with RA.

One study(26) found that patients reported substantial hospital costs, which were even greater in patients with high disease activity (i.e. severe disease) compared with those with low disease activity or remission. These extra costs, mainly driven by non-drug resource use, emphasize the importance of treating early and effectively to achieve a low disease activity score (as measured by DAS28) and consequently reduce economic burden.

RA also has a significant impact on indirect costs. Levels of absenteeism and presenteeism as a cost burden for the economy was the focus for Leggett et al. (2016) and Leggett et al. (2017)(27, 28). Using the BSR Biologics Register for RA (BSRBR-RA), both showed improvements after 1 year of treatment with advanced therapy. Even then, however, absenteeism and presenteeism still persisted; levels of the former only decreased from 21% to 17%. This persistence is not surprising given that after 2 years of early active treatment in patients with new-onset RA, a substantial portion had unacceptable pain despite inflammation control.(29) However, Leggett et al. did find that patients who experienced a good or moderate response to advanced therapy at 6 months were significantly more likely (2.8 times more likely; p < 0.05) to have no presenteeism at 1 year.

In a cross-sectional study by Houssien et al. (2017)(30), patients with RA who screened positive for depression had a significantly greater risk of work disability than patients without depression, even after adjusting for available confounders (age, sex, disease duration, Heath Assessment Questionnaire – Disability Index [HAQ-DI] and DAS28 scores). The magnitude of association observed was even greater than that seen between physical function and work. Therefore, there is the need to target depression; otherwise, both the patient and society are likely to face substantial economic costs.

### 1.2.2 Humanistic burden of disease

Results from these studies were consistent, finding that patients with RA have substantial disease burden in terms of disability, fatigue, pain, and comorbidities.

In a UK study of 2,045 patients with RA, a significant relationship was found between increasing disease activity and increased disability (higher HAQ-DI), disability progression, and a need for major orthopaedic surgery(18). However, another study found that despite patients reporting being in a ‘patient acceptable symptom state’, many still had high levels of disability (on HAQ-DI), depression, fatigue, pain, high DAS28 scores, and high swollen joint counts and tender joint counts(31). Nearly half of patients (47%) studied from the Early Rheumatoid Arthritis Study and Early Rheumatoid Arthritis Network had moderate DAS28 scores; however, despite significant disability, progression, and a need for major orthopaedic surgery, these patients would not be eligible for biological disease-modifying anti-rheumatic drugs (bDMARDs) according to National Institute for Health and Care Excellence guidance(18).

One study reported that as many as 89% of patients with RA experienced chronic fatigue. Among those, 98% said it affected their lives: 71% of unemployed, working-age patients said that chronic fatigue contributed to not being unable to work, and 24% said they had to change jobs as a result of their chronic fatigue(32). In a study specifically focused on patients with severe RA, patients with persistent fatigue reported significantly more overall pain, significantly more recent and current pain intensity, and significantly higher levels of depression than patients with no fatigue(33). There was no significant difference between groups in self-efficacy, and both groups experienced poor sleep quality. Despite having good disease response to tumour necrosis factor (TNF) inhibitors, the persistent fatigue group had significantly higher rheumatoid factor incidence, significantly higher DAS28 scores, significantly longer durations of early morning stiffness, and significantly lower incidence of ever-failing conventional synthetic DMARDs (csDMARDs) than the patients with no fatigue(33).

In a study of the BSRBR-RA database, patients with RA reported moderate to severe pain (on the 36-item Short Form Health Survey) at baseline, with mean pain scores >2 standard deviation (SD) higher than the UK general population(34). Although patients improved on treatment after 1 year, pain scores remained >1 SD worse than the normal population level.

Peterson et al. (2016)(35) observed that 38.4% of patients with RA reported moderate or extreme anxiety/depression as their current health state. These patients had greater work impairment, higher disability (based on HAQ-DI scores), higher DAS28 scores, and a higher likelihood of being dissatisfied with their current treatment compared with patients without depression or anxiety.

Patients with RA were also found to experience significant comorbidities, with as many as 70% experiencing comorbidities in a study of patients with severe RA⸺mostly infection, cardiovascular disease, and depression(36). They found that in patients with a European League Against Rheumatism (EULAR) moderate response or better, 82% had comorbidities; while in patients with a EULAR good response, 62% had comorbidities. However, no statistically significant correlation was found between EULAR response and comorbidity. Therefore, regardless of response to treatment and current disease activity, these patients are still likely to experience a number of comorbidities.

### 1.2.3 Caregiver burden of disease

No studies on caregiver burden were identified in the moderate to severe or severe populations.

### 1.2.4 Real-world clinical outcomes

Because there was a significant amount of heterogeneity between the studies and the evidence presented, it is difficult to draw any direct comparisons between the studies. However, it is clear from the data that there remains a significant proportion of patients who do not achieve disease remission and who retain some level of disease activity with current treatment patterns. Also of note, studies in severe patients were more focused on advanced treatments, whereas the moderate to severe studies included a mix of csDMARDs and bDMARDs, whether with TNF inhibitors or biologics with an alternative mechanism of action. The difference in focus between the studies of difference populations reflects the increased restriction around accessing advanced therapies for patients, which could explain some of the potential unmet need in this population.

Table S4. Summary of real-world clinical outcomes in moderate to severe and severe RA

| Study | Study type [N] | Treatment | Remission | Response | Steroid use | DAS28 | Other |
| --- | --- | --- | --- | --- | --- | --- | --- |
| Moderate to severe RA | | | | | | |  |
| Bergstra, 2018(12) | Retrospective database study [N=20,379] | Standard practice in the included countries (including csDMARDs and bDMARDs) | 26.0% | – | – | **Mean final DAS28:** 3.9 | – |
| Benson, 2018(37) | 6-month retrospective medical chart review [N=111] | TNF-α inhibitor | 17% | **Moderate response:** 14% | – | **Mean change in DAS28:** 2 | – |
| Buch, 2013(38) | Prospective audit [N=1,158] | – | 6 months: 39% | **Moderate or good EULAR:** 68% | – | **DAS28, mean** Baseline: 4.7 (SD 1.6) 6 months: 3.1 (SD 1.4) | – |
| Buch, 2015(39) | Prospective audit [N=1,158] | – | 6 months: 42% 12 months: 54% | **Low disease activity:** 6 months: 15% 12 months: 15% **Moderate disease activity:** 6 months: 31% 12 months: 25% **High disease activity:** 6 months: 12% 12 months: 6% | – | – | – |
| Buch, 2015^a(20)^ | Prospective audit [N=1,158] | 70% DMARDs (45% single, 24% two and 1% three DMARDs)  0% biologics | – | – | 74% | – | – |
| Byrne, 2017(40) | Retrospective case note review [N=132] | TCZ either alone (34.6%) or in combination with MTX (42.1%) or other DMARDs (23.3%) | – | – | 23% | – | **Discontinued treatment:** 33% |
| Choy, 2012(21) | Retrospective cohort [N=1,194] | Biologics | – | – | – | **DAS28, mean (range):**  Baseline: 6.2 (1.8-8.6) |  |
| Emery, 2015(19) | Retrospective medical chart review [N=111] | Biological therapy | 44.5% **[Remission maintained throughout follow-up:** 41.3%**]** | – | – | – | – |
| Gwinnutt, 2017(41) | Database study [N=602] | Standard practice | NR | NR | 28.1% | – | – |
| Hughes, 2017(36) | Retrospective patients note review [N=132] | Biologics | – | **EULAR moderate:** 70% **EULAR good:** 26% **No response:** 30% | – | – | – |
| Ismajli, 2017(42) | Retrospective study [N=356] | RTX (monotherapy: 25.3%; with prednisolone: 7.3%; with DMARD: 67.7%) | 43.9% | **Disease activity:** Low: 13.2% Moderate: 32.8% High: 7.4% | 7.3% | – | – |
| McWilliams, 2016(34) | 1-year retrospective database study [N=15,627] | TNF-α inhibitor cohort (n=11,995) | 17% | **Non-response:** 17% **EULAR moderate:** 54% **EULAR good:** 29% | 44% | – | – |
|  |  | Non-biological cohort (n=3,632) | 18% | **Non-response:** 48% **EULAR moderate:** 29% **EULAR good:** 23% | 23% | – | – |
| Mian, 2016(17) | Cross-sectional study [N=1,324] | Standard practice | 1996-7: 8% 2001-3: 9% 2012-14: 28% | **Moderate disease activity:** 1996-7: 38%% 2001-3: 37% 2012-14: 41% | 1996-7: 12% 2001-3: 23% 2009-10: 10 2012-14: 12% | – | – |
| Ogdie, 2018(43) | Database study [51,762] | DMARD (n=31,336) | NR | NR | 43.0% | – | – |
|  |  | No DMARD (n=20,426) | NR | NR | 22.6% | – | – |
| Tan, 2017^a^(23) | Prospective audit [N=1,571] | Dual csDMARD or biological therapy | 12 months: 53.6% 24 months: 25% | – | – | – | – |
| Yousif, (2016)^a^(22) | Prospective audit [N=1,571] | csDMARD | 12 months: 68.4% | – | **Baseline:** Intramuscular: 51.0% Oral: 9.1%  Intra-articular: 4.6% **12 months:** Intramuscular: 66.7% Oral: 10.2% Intra-articular: 0.9% [not mutually exclusive] | **DAS28, mean** Baseline: 4.9 12 months: 2.4 | **Patients achieving target at 12 months:** 68.4% |
| Yusof, 2016(44) | Retrospective observational study [N=61] | Rituximab (67% received concomitant DMARD) | – | **Moderate-good EULAR:** Cycle 1: 87% Cycle 2: 91% Cycle 3: 86% | – | **DAS28, median reduction (IQR):** 6 months: 2.1 (0-3.4) | – |
| Severe RA | | | | | | |  |
| Ismajli, 2017(45) | Retrospective study [N=40] | RTX | Remission or low disease activity: 1/3 | Moderate disease activity: >1/2 | – | **DAS28, change in score, median:** **RTX, first treatment:** Group A: -1.83 Group B: -1.07 **RTX, re-treatment:** Group A: -2.19 Group B: -1.29 | – |
| Kearsley-Fleet (2018)(46) | Registry study [N=13,502] | First-line TNF-α inhibitor | – | **bDMARD refractory:** 6.4% | – | – | Median time from first TNF-α inhibitor to bDMARD refractory disease: 7.9 years (95% CI, 5.7-10.0) |
| Leggett, 2016 and Leggett, 2017(27, 28) | Retrospective database study [N=263] | Biologics | – | **EULAR good/moderate:** 81% | – | – | **HAQ-DI, change at 6 months, mean (SD):** -0.35 (0.52) **EQ-5D, change at 6 months, mean (SD):** 0.13 (0.28) |
| Minnock, 2015(33) | Cross-sectional observational study [N=64] | TNF-α inhibitor | 6 months: 34.4% | **DAS28 >3.2 at 6 months:** 25% | – | **DAS28 at 6 months, mean (SD):** Persistent fatigue group: 3.3 (1.1) No fatigue group: 2.4 (0.7) | – |
| Nolkha, 2018(47) | Retrospective, single-centre analysis [N=220] | Etanercept | – | **EULAR response (6 months):** Good: 38% Moderate: 37% No response: 25% | – | **DAS28 score, mean:** Baseline: 6.0 (SD 3.1) Change at 6 months: -1.5 (95% CI, 1.27 to -1.33) | – |
| Oldroyd, 2018(48) | Registry study [N=1,629] | bDMARD experienced: 1,371 bDMARD naive: 258 | – | – | – | – | – |
| Shipa, 2017(49) | Retrospective observational study [N=422] | TNF-α inhibitor (n=211) | – | **At least a moderate EULAR response:** 35% | – | – | – |
|  |  | Biologics (RTX, ABT, TCZ) (n=211) | – | **At least a moderate EULAR response:** 70% (RTX: 75%; ABT: 65%; TCZ: 70%) | – | – | – |
| ^a^Three articles reporting the same study.  ABT, abatacept; bDMARD, biological disease-modifying anti-rheumatic drug; csDMARD, conventional synthetic disease-modifying anti-rheumatic drug; DAS28, 28-joint disease activity score; DMARD, disease-modifying anti-rheumatic drug; EQ-5D, EuroQol 5-dimensions; EULAR, European League Against Rheumatism; HAQ-DI, Health Assessment Questionnaire Disability Index; IQR, interquartile range; MTX, methotrexate; NR, not reported; RA, rheumatoid arthritis; RTX, rituximab; SD, standard deviation; TCZ, tocilizumab; TNF-α, tumour necrosis factor alpha. | | | | | | | |
