# Supplementary material for: Targeted literature review of current treatments and unmet need in moderate rheumatoid arthritis in the United Kingdom
Source: Rheumatology (Oxford). 2021 Jun 3;60(11):4972–81. doi: 10.1093/rheumatology/keab464 (PMC8566217; doi:10.1093/rheumatology/keab464)
Supplement: keab464_Supplementary_Data [file keab464_supplementary_data.zip › keab464-suppl_data/Supplementary References.docx]

25. Kirkham B, Chan E, Vincent A, Elliott A. Real-life cost of uk healthcare resource for patients with rheumatoid arthritis, comparing high and low/remission disease states. Arthritis and Rheumatology. 2016;68:2916-7.

26. Leggett S, Hyrich KL, Lunt M, Walker-Bone K, Verstappen SMM. The importance of achieving clinical response to treatment and changes in physical ability and quality of life on worker productivity outcomes in rheumatoid arthritis: Results from the British society for rheumatology biologics register. Arthritis and Rheumatology. 2016;68:29-31.

27. Leggett SA, Hyrich K, Lunt M, Walker-Bone K, Verstappen SMM. The importance of achieving clinical response to treatment and changes in physical ability and quality of life on worker productivity outcomes in rheumatoid arthritis: Results from the british society for rheumatology biologics register for rheumatoid arthritis. Rheumatology (United Kingdom). 2017;56:ii113.

28. Olofsson T, Wallman J, Jöud A, Schelin M, Ernestam S, van Vollenhoven R, et al. OP0133 Unacceptable, refractory pain despite inflammation control in early rheumatoid arthritis and its relation to treatment strategy: results from the randomised controlled swefot trial. BMJ Publishing Group Ltd; 2018.

29. Houssien A, Norton S, Nikiphorou E, Matcham F, Galloway J. The association between work disability and mental health in rheumatoid arthritis. Annals of the Rheumatic Diseases. 2017;76:805-6.

30. Gwinnutt J, Hyrich K, Lunt M, Barton A, Verstappen S. High disease activity and disability at one year in two clusters of patients with rheumatoid arthritis defining themselves as in an acceptable state at treatment initiation. Annals of the Rheumatic Diseases. 2018;77:71.

31. National Rheumatoid Arthritis Society. . Invisible disease: Rheumatoid arthritis and chronic fatigue survey 2014 [Available from: <https://www.nras.org.uk/invisible-disease-rheumatoid-arthritis-and-chronic-fatigue-report>.

32. Minnock P, Veale DJ, Bresnihan B, FitzGerald O, McKee G. Factors that influence fatigue status in patients with severe rheumatoid arthritis (RA) and good disease outcome following 6 months of TNF inhibitor therapy: a comparative analysis. Clinical Rheumatology. 2015;34(11):1857-65.

33. McWilliams DF, Walsh DA. Factors predicting pain and early discontinuation of tumour necrosis factor-?-inhibitors in people with rheumatoid arthritis: Results from the British society for rheumatology biologics register. BMC Musculoskeletal Disorders. 2016;17(1).

34. Peterson S, Li N, Blackburn S, Kielar D. Anxiety and depression among rheumatoid arthritis patients, and association with clinical measurements and patient reported disability and treatment satisfaction. Annals of the Rheumatic Diseases. 2016;75:697.

35. Hughes CD, Gullick N. Does comorbidity adversely impact on treatment response in patients with rheumatoid arthritis. Annals of the Rheumatic Diseases. 2017;76:1154-5.

36. Benson R, Kapur D, Goodson N, Abernethy R, Barnes T. Biologic monotherapy in rheumatoid arthritis: A retrospective observational study of real life practice in the North West. Rheumatology (United Kingdom). 2018;57:iii197.

37. Buch M, O'Reilly D, Sheeran T, Keidel S, Emery P. Compliance with treat to target recommendations and its impat on control of rheumatoid arthritis - interim results of a UK multicentre audit. EULAR2013.

38. Buch M, O'Reilly D, Sheeran T, Keidel S, Emery P. Compliance with treat to target recommendations on rheumatoid arthritis and patient outcomes - interim results of a UK multicentre study. BSR2015.

39. Byrne E, Mark P, Khalid S, Kuet KP, Kilding R, Graves K, et al. Tocilizumab for the management of rheumatoid arthritis: Discontinuation due to inefficacy and toxicity-experience from a large teaching hospital. Annals of the Rheumatic Diseases. 2017;76:847.

40. Gwinnutt JM, Symmons DPM, MacGregor AJ, Chipping JR, Marshall T, Lunt M, et al. Twenty-Year Outcome and Association Between Early Treatment and Mortality and Disability in an Inception Cohort of Patients With Rheumatoid Arthritis: Results From the Norfolk Arthritis Register. Arthritis and Rheumatology. 2017;69(8):1566-75.

41. Ismajli M, Ionescu R, Moore S, Leandro M. Long-term use of rituximab in rheumatoid arthritis: 17 years follow-up. Rheumatology (United Kingdom). 2017;56:ii142.

42. Ogdie A, McGill NK, Shin DB, Takeshita J, Love TJ, Noe MH, et al. Risk of venous thromboembolism in patients with psoriatic arthritis, psoriasis and rheumatoid arthritis: a general population-based cohort study. Eur Heart J. 2018;39(39):3608-14.

43. Yusof MYM, Iqbal K, Emery P, Dass S, Kelly C. Efficacy and safety of rituximab in patients with rheumatoid arthritis-related bronchiectasis (RA-BR): Results from a multicentre cohort. Annals of the Rheumatic Diseases. 2016;75:996.

44. Ismajli M, Ionescu R, Moore S, Cambridge G, Leandro M. Optimization of rituximab therapy in rheumatoid arthritis: Second cycle at 6 months and predictors of improved response. Rheumatology (United Kingdom). 2017;56:ii203-ii4.

45. Kearsley-Fleet L, Davies R, De Cock D, Watson KD, Lunt M, Buch MH, et al. Biologic refractory disease in rheumatoid arthritis: results from the British Society for Rheumatology Biologics Register for Rheumatoid Arthritis. Annals of the rheumatic diseases. 2018;77(10):1405-12.

46. Nolkha N, Sheeran T, Venkatachalam S. Drug survival and efficacy of abatacept in rheumatoid arthritis patients in routine care-7 year experience from a single centre in the United Kingdom. Annals of the Rheumatic Diseases. 2018;77:959-60.

47. Oldroyd AGS, Symmons DPM, Sergeant JC, Kearsley-Fleet L, Watson K, Lunt M, et al. Long-term persistence with rituximab in patients with rheumatoid arthritis. Rheumatology (United Kingdom). 2018;57(6):1089-96.

48. Shipa M, Cicco MD, Balogh E, Mian A, Mukerjee D, Roussou E. Tailoring second-line biologic therapy in rheumatoid arthritis: New findings on the usefulness of antibody status to optimise drug selection. Arthritis and Rheumatology. 2017;69.
